# Opportunities and Challenges of California’s Fruit and Vegetable Electronic Benefit Transfer Pilot Project at Farmers’ Markets: A Qualitative Study with Supplemental Nutrition Assistance Program Shoppers and Farmers’ Market Staff

**DOI:** 10.3390/nu16193388

**Published:** 2024-10-05

**Authors:** Carolyn Chelius, Ron Strochlic, Sridharshi C. Hewawitharana, Wendi Gosliner

**Affiliations:** Nutrition Policy Institute, Division of Agriculture and Natural Resources, University of California, Oakland, CA 94607, USA; cchelius@unc.edu (C.C.); shewawitharana@ucanr.edu (S.C.H.); wgosliner@ucanr.edu (W.G.)

**Keywords:** nutrition policy, SNAP, nutrition incentive programs, GusNIP, farmers’ markets

## Abstract

Background/Objectives: Nutrition incentive programs can increase access to fresh fruits and vegetables and improve food security among Supplemental Nutrition Assistance Program (SNAP) participants and others in the United States. This qualitative study explored SNAP participants’ and farmers’ market staff’s perceptions of and experiences with a pilot supplemental benefit program offered at seven farmers’ markets in 2023 as part of the California Fruit and Vegetable Electronic Benefit Transfer Pilot Project. The pilot introduced specific characteristics that differ from more traditional nutrition incentive program dollar-for-dollar match incentive models, particularly the traditional model operating in California. Specific differences included the following: (1) offering a monthly USD 60 supplemental benefit that could be redeemed in a single shopping trip; (2) providing the supplemental benefit as a match that could be spent on any SNAP-eligible item in any retail location (rather than solely on fruits and vegetables at the farmers’ market). Methods: We conducted a qualitative, cross-sectional study including seven focus groups with 40 SNAP shoppers and six focus groups with 14 farmers’ market staff at six pilot-participating farmers’ markets in California. All focus groups were conducted by trained research staff and were recorded, transcribed, and analyzed using the immersion crystallization method. Results: Our findings include that shoppers appreciated several features of the pilot, particularly the ability to obtain an entire month’s supplemental benefit during one shopping trip and the ability to spend the benefit earned on any SNAP-eligible item at any SNAP retailer. Farmers’ market staff appreciated that the pilot benefited shoppers but found it difficult to manage due to staff and shopper confusion about the program, increased program utilization, subsequent long lines, and the spending of the earned incentives at other SNAP retailers. Both shoppers and staff reported that the program was difficult to understand. Conclusions: These findings can inform future nutrition incentive program designs to benefit SNAP participants while offering sustainable models for farmers’ markets.

## 1. Introduction

Most U.S. adults do not consume the recommended quantities of fruits and vegetables, despite evidence linking fruit and vegetable consumption to improved health and reduced risk of multiple chronic diseases, including cardiovascular disease and diabetes [1]. Disparities in fruit and vegetable consumption persist, as Americans with lower incomes consume fewer fruits and vegetables than those with higher incomes [2]. One frequently cited barrier to produce intake is the high per-calorie cost of fresh fruits and vegetables relative to the lower per-calorie cost of nutrient-poor foods [3,4].

The Supplemental Nutrition Assistance Program (SNAP) is the most significant source of food assistance in the U.S., providing USD 107 billion in benefits to 42 million low-income Americans in 2023 [5]. SNAP funds are distributed through electronic benefit transfer (EBT) “debit” cards that can be used to make purchases at participating retailers. SNAP funds can be spent on most foods and beverages, excluding alcohol and certain prepared foods, like those that are hot at the point of sale [6]. Unlike poverty alleviation programs in many other peer nations, the U.S. safety net system primarily focuses benefits on in-kind food aid, making the federal food program policies critical for people experiencing food insecurity and economic hardship [7].

Nutrition incentive programs provide additional funds for fruits and vegetables purchased using SNAP benefits, frequently as dollar-for-dollar matched funds [8]. These programs were first launched in 2005 as a strategy to make healthy foods more affordable for consumers with low incomes while supporting local agriculture, which tends to be organic or pesticide-free, thus also supporting more sustainable food systems [9,10]. Nutrition incentive programs have expanded significantly since 2005: in 2023, over 1500 unique SNAP retailers, including farmers’ markets and grocery stores, offered nutrition incentive programs [11]. The federal Gus Schumacher Nutrition Incentive Program (GusNIP), established in 2019, funds nutrition incentive programs, as do several private foundations, nonprofit organizations, and other sources [8]. While program designs vary, most provide a dollar-for-dollar match on SNAP funds spent on fruits and vegetables, up to a per-visit maximum of typically USD 5–20 [11]. While nutrition incentive programs are now offered at a variety of retail outlets in the U.S., they are still most commonly offered at farmers’ markets [11].

A significant body of research on U.S. nutrition incentive programs finds that program participants are grateful for the programs, appreciating the extra funds provided to support their household food purchases [12,13,14]. In terms of outcomes, studies primarily focus on changes to shopper produce purchases and consumption [15]. Some studies have found associations between incentive programs and increased participant produce intake [16,17,18,19]. Other studies have found associations with increased produce purchases and/or indicators of food security but not consumption [12,20]. Heterogenous incentive designs can make it challenging to determine which programmatic features are most effective [17].

Fewer studies have analyzed program operator perspectives. An evaluation of a nutrition incentive program at farmers’ markets in New York City found that market managers reported that the program attracted new shoppers to farmers’ markets and that farmers’ market vendors reported they made more money and had more repeat shoppers with the program [21]. A study in Maryland found that farmers’ market managers reported that the nutrition incentive program provided benefits to shoppers and vendors through increased food access and sales, thereby aligning with the market’s mission to increase access to high-quality food [22].

The largest nutrition incentive program in California is the California Nutrition Incentive Program (CNIP), which has operated since 2017. CNIP is funded as a partnership between federal GusNIP funds and state and private funds [23]. The majority of sites that administer CNIP are farmers’ markets [23]. CNIP program designs vary by site, but most provide a dollar-for-dollar match on SNAP funds spent at the market, up to a maximum of typically USD 5–20 per shopping trip. The match funds can only be used to purchase fruits and vegetables and other “specialty crops”, such as herbs and seedlings. Evaluations of CNIP indicate that the program is well received by participants, who report an increased ability to purchase high-quality fresh produce and eat healthily [12]. CNIP program participants have also reported that the program influences their decision to shop at farmers’ markets [12].

In February 2023, the California Department of Social Services (CDSS) launched the California Fruit and Vegetable EBT Pilot Project at seven farmers’ markets to test alternative methods for administering nutrition incentive programs. This study assessed shopper and staff perceptions of the program, hereafter referred to as the “pilot”. In particular, the study captured shopper and staff perceptions of the benefits and drawbacks of the pilot compared to more traditional CNIP—hereafter referred to as “traditional CNIP”. The pilot differed from traditional CNIP in two primary ways:

The pilot offered a monthly USD 60 maximum match that could be redeemed in a single visit or multiple visits during the same month, whereas traditional CNIP offers a variable weekly maximum match of USD 5–20.

The pilot benefit was earned when SNAP funds were spent on fruits and vegetables and was applied directly to shoppers’ EBT cards for purchasing any SNAP-eligible item at any retail location. Traditional CNIP incentives were earned when SNAP funds were spent at the farmers’ market and could only be redeemed for fruits and vegetables at the farmers’ market.

These differences make this supplemental benefit program distinct from other similar programs in California and across the U.S. According to a recent evaluation of federally funded nutrition incentive programs, 91% of farm-direct sites (e.g., farmers’ markets and farm stands) require shoppers to spend their SNAP dollars at the market to earn incentive funds for purchasing fresh fruits and vegetables [11]. The only other program that reverses this model is the Healthy Incentive Program (HIP) in Massachusetts [24]. The HIP shares several programmatic features with the pilot studied here, including the ability to earn the supplemental benefit on the purchase of fruits and vegetables, which is credited to participants’ SNAP accounts and can be used to purchase any SNAP-eligible item. Our study is therefore one of the few to evaluate this model of SNAP incentive programs. This study aims to evaluate the experiences of shoppers and market managers with the pilot and assess its perceived impact on shoppers’ use of farmers’ markets and their produce purchasing and consumption behaviors, as well as market operators’ sales and management processes. The study findings can inform future nutrition incentive programs.

## 2. Materials and Methods

### 2.1. Study Sample

This study included SNAP shoppers and farmers’ market staff from six of seven farmers’ markets that implemented the pilot. The markets, which were located in Northern and Southern California, implemented the program during the period 25 February–4 April 2023. All markets participated in CNIP before implementing the pilot, with 3 offering a USD 10 per visit (weekly) maximum match and 3 offering a USD 15 maximum match. One market offered an unlimited match before and during the pilot, which meant that shoppers did not experience a change in benefits with the pilot program compared to CNIP. This market was therefore excluded from this study.

### 2.2. Participants and Recruitment

#### 2.2.1. SNAP Shoppers

##### Recruitment and Screening

SNAP shoppers were recruited to participate in this study during the period June–July 2023. At five markets, a member of the research team recruited shoppers in person. All shoppers approaching the EBT booth at participating farmers’ markets were asked if they had used the pilot previously. Those answering yes (i.e., for whom it was not their first time using the program) were invited to provide their contact information so that the research team could contact them about this study. Due to language barriers at the sixth market, market staff conducted recruitment and provided the research team with contact information for interested shoppers.

Interested SNAP shoppers were contacted via text or email and invited to complete a brief online screener to establish their study eligibility. Shoppers were deemed eligible if they were over 18, spoke English or Spanish, were a member of a household receiving SNAP benefits, and had shopped at a pilot-participating market during the preceding month. Eligible shoppers were invited to electronically sign up to attend a virtual focus group after completing the screener.

##### Data Collection

Seven focus groups were held via Zoom between June and August 2023, of which six were conducted in English and one in Spanish. Three members of the research team participated in the focus group discussions as facilitators and/or notetakers. Each focus group included one facilitator and one notetaker. Participants were sorted into focus groups based on the market from which they were recruited and their availability. Only one focus group included shoppers from multiple markets. Focus group participants received a USD 50 gift card in appreciation for their participation.

A semi-structured focus group guide was developed by the research team to elicit shopper perceptions of and experiences with the pilot, including their perceived changes in shopping habits, diet quality, and food security (Table 1). The focus group guide was reviewed by staff at the CDSS and the Ecology Center (pilot administrator) and was revised based on feedback. All focus groups were conducted via Zoom.

#### 2.2.2. Farmers’ Market Staff

##### Recruitment and Screening

All staff at the six pilot-participating farmers’ markets included in this study were invited to participate in focus groups. The research team connected with one key staff member, typically a manager, at each market, who recruited additional staff members to participate.

##### Data Collection

One research team member conducted focus groups with market staff between July and August 2023. A total of five focus groups were conducted with staff: four with staff from each individual market and one with staff from two markets operated by the same organization. Focus group questions explored staff members’ experiences operating the pilot; their perceptions of benefits and challenges for staff, shoppers, and vendors; and recommendations for improvements. The focus group guide was reviewed by staff at the CDSS and the Ecology Center and was revised based on feedback. All focus groups were conducted via Zoom (Table 2).

#### 2.2.3. Ethical Approval

The Institutional Review Board of the University of California, Davis approved this study.

### 2.3. Data Analysis

English-language focus group transcripts were transcribed using Otter.ai (https://otter.ai/. Access date 21 August 2023) and reviewed by a member of the research team for errors. The focus group conducted in Spanish was transcribed and translated using a professional translation service. Vocal disfluencies were removed from all transcripts.

The research team used the immersion crystallization method of qualitative data analysis [25]. Two researchers immersed themselves in the data by reading all transcripts. Both researchers developed an initial codebook using a deductive approach and jointly coded one shopper focus group and one staff focus group transcript line-by-line to ensure consistency in the application of codes. Additional codes were subsequently added using an inductive approach. One researcher then coded the remaining transcripts, and the other reviewed the first coder’s work, resolving coding discrepancies via consensus. The same set of codes was used for both staff and shopper transcripts, given thematic overlap, and to facilitate comparing and contrasting findings between the two stakeholder groups. After coding all staff and shopper focus groups, the two researchers engaged in the crystallization phase of analysis, articulating and reflecting upon major themes and synthesizing them into categories. The full research team met weekly to further discuss and refine themes. All coding and data analysis was conducted using ATLAS.ti (Version 24.0.1) [26].

## 3. Results

### 3.1. SNAP Shopper Focus Groups

**Figure 1:**
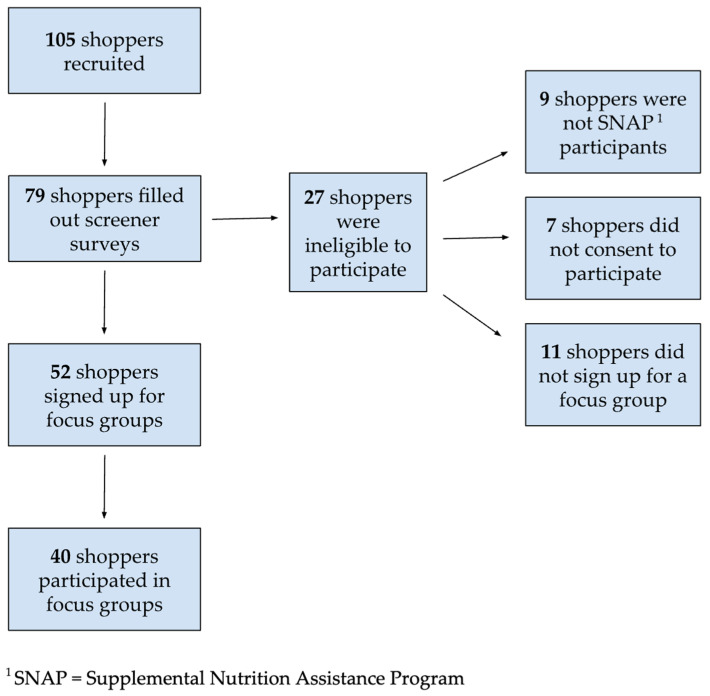
Sample selection flow chart for focus groups with Supplemental Nutrition Assistance Program shoppers participating in the Fruit and Vegetable Electronic Benefit Transfer Pilot Project in California, 2023.

Of the 105 shoppers initially invited to participate in this study, 79 filled out eligibility screener surveys (Figure 1). Twenty-seven respondents were ineligible to participate, as they were not SNAP participants (*n* = 9), did not provide consent to participate (*n* = 7), or did not sign up for a focus group (*n* = 11). Fifty-two shoppers were eligible to participate and signed up for focus groups. Of those, 40 participated in focus groups (Table 3). The number of participants in each focus group ranged from 1 to 10, with an average of 5.7. Focus groups included a mix of shoppers who had experience using traditional CNIP and those for whom the pilot was their first experience with match incentive/supplemental benefit programs.

The majority of shopper participants (65%) were female, with individuals identifying as White representing the largest ethnic group (42.5%), followed by Latino/a and Asian shoppers (both 25%) (Table 4).

### 3.2. Farmers’ Market Staff Focus Groups

Five focus groups were conducted with a total of 14 farmers’ market staff. Participation ranged from 1 to 5 staff members per focus group (Table 5).

### 3.3. Key Findings

Fourteen key findings emerged from the focus groups with shoppers and farmers’ market staff (Table 6). Findings are presented in three categories – shopper-only findings, shopper- and staff-shared findings, and staff-only findings – as there was overlap in some shopper and staff findings, while other findings emerged only from one of the two populations. Key findings reflect themes that arose in nearly all focus groups and were typically reported by multiple participants per group.

#### 3.3.1. Key Findings from Shopper Focus Groups

Shoppers overwhelmingly reported positive perceptions of the pilot and reported preferring many aspects of the pilot structure over traditional CNIP. They appreciated being able to receive the full benefit in one monthly visit rather than weekly, which was particularly helpful for disabled shoppers or those with dependents, for whom traveling to the market each week was difficult. Shoppers at markets previously offering a USD 10 per visit match also greatly appreciated the USD 60 monthly benefit. The higher benefit amount, coupled with the ability to earn and redeem the entire benefit in one visit, allowed shoppers to purchase more—and often more expensive—fruits and vegetables. Shoppers also greatly appreciated the option of keeping the entire benefit on their EBT card and being able to spend it on any SNAP-eligible item at any retailer rather than only on produce at the farmers’ market. In that regard, shoppers noted that the supplemental benefit felt like “free money”. Most shoppers reported spending the benefit at other retailers on staple items that were not available at or more affordable than at farmers’ markets. Nonetheless, some reported purchasing non-produce items such as eggs and honey at the farmers’ market. Shoppers cited confusion regarding several aspects of the pilot, including the requirement to have a minimum EBT balance to obtain the maximum match since the supplemental benefit was credited directly to their EBT cards, and they were not required to spend those funds at the farmers’ market. Additionally, since news of the pilot was often spread via word-of-mouth, many shoppers understood the program as USD 60 of “free” money and were disappointed to learn of the requirement to spend EBT funds to earn the supplemental benefit. An additional source of confusion—and in some cases, mistrust—for shoppers familiar with the previous approach was that the supplemental benefit was electronically credited to shoppers’ EBT cards, whereas traditional CNIP benefits are provided in the form of physical tokens or scrip, which offer tangible proof of having received the benefit. Shoppers reported logistical challenges as well, including receiving as many as 60 one-dollar tokens, given the lack of larger denominations.

#### 3.3.2. Key Findings from Shopper and Staff Focus Groups

Both shoppers and market staff reported that the pilot attracted new SNAP shoppers to participating farmers’ markets, noting that increased attendance created long lines, which were exacerbated by the time required to explain the pilot to shoppers. Staff at some markets reported queues of up to three hours before the market opened. Staff and shoppers reported that the pilot was difficult to understand, which was a more significant issue for those familiar with traditional CNIP than new shoppers with no previous frame of reference. Staff and shoppers reported that confusion was exacerbated for shoppers who spoke languages other than English.

#### 3.3.3. Key Findings from Staff Focus Groups

Staff appreciated that the pilot offered shoppers a higher benefit amount and greater flexibility regarding where shoppers could spend those funds. Additionally, staff noted a “workaround” allowing them to provide the full benefit to shoppers with an EBT balance of less than USD 60. That was accomplished by repeatedly applying the benefit in smaller increments until the full USD 60 was reached—an approach that is not possible with traditional CNIP. Staff felt that vendors benefitted from increased shopper attendance and higher benefits; however, they expressed concerns regarding potential negative impacts on non-produce vendors, given shoppers’ tendencies to purchase fruits and vegetables at the farmers’ market and spend the supplemental benefit at other retailers. Under traditional CNIP, shoppers are required to purchase tokens that could be spent on anything sold at the farmers’ market and spend earned incentives only on produce at the farmers’ market. Staff appreciated that the pilot offered shoppers increased choice by allowing them to spend the supplemental benefit at grocery stores; however, they expressed concerns regarding reduced farmers’ market sales. Staff at every market discussed technological and logistical challenges, including insufficient point-of-sale devices and market scrip to meet increased demand, EBT mischarges, and the inability to check shopper incentive balances. Staff also reported that the pilot required significantly more time to process paperwork than traditional CNIP and subsequently required a greater number of more highly trained staff. Several staff noted that the increased stress and workload associated with the pilot made it more difficult to recruit and train staff, including volunteers. Staff also reported that the quality of their interactions with shoppers was diminished due to increased shopper attendance and more time being required to explain the program, further contributing to job dissatisfaction. They expressed discomfort with having to ask questions regarding the amount of produce shoppers planned to purchase, which was perceived as invasive.

## 4. Discussion

This study explores shopper and farmers’ market staff perceptions of a new approach for delivering nutrition incentives to SNAP shoppers at farmers’ markets in California: the California Fruit and Vegetable EBT Pilot Project. This study found that shoppers appreciated several features of the pilot, particularly the ability to obtain the full benefit in one monthly visit. Shoppers and staff appreciated higher benefit amounts and the flexibility to spend the benefit on any SNAP-eligible item at any SNAP retailer. Shoppers and staff cited difficulties understanding the pilot and concerns regarding long lines. Staff also reported technological challenges, reduced quality of interactions with shoppers, and increased stress and workload.

The pilot model contained several features which, our findings suggest, if expanded, would largely benefit SNAP shoppers but could have mixed impacts on farmers’ markets. The features include the following: (1) larger monthly benefits for shoppers at some markets; (2) the ability of shoppers to obtain the entire monthly benefit at once; (3) the delivery of benefits as an automatic rebate applied to a shopper’s EBT card; and (4) the ability for shoppers to spend supplemental benefits on any SNAP-eligible items anywhere that accepts SNAP. The following section discusses what we learned about each of these features and their implications if the pilot model were to be scaled up in California or other jurisdictions.

### 4.1. Larger Benefits for Shoppers

The pilot provided a USD 60 per month incentive, which is higher than the amounts offered by many nutrition incentive programs, including in California. Shoppers reported that these increased funds were beneficial in allowing them to purchase more and higher quality produce. This finding aligns with research demonstrating that SNAP shoppers appreciate nutrition incentive programs for the financial and nutritional benefits that they provide [17] and that they value increases in incentive match maximums and the ability to purchase more produce [27]. Research also demonstrates that higher incentives are associated with increased produce purchases by SNAP shoppers [28] and improvements in some measures related to food security [12].

Farmers’ market staff also appreciated the increased benefits. They valued the ability to provide shoppers with increased funds for purchasing produce and felt that the program attracted new shoppers to the market. Previous research has demonstrated market staff appreciate nutrition incentive programs’ role in increasing SNAP shoppers’ access to high-quality food and improving food security and that nutrition incentive programs benefit farmers’ markets and vendors by attracting new and repeat shoppers [21,22].

### 4.2. Ability for Shoppers to Obtain the Entire Monthly Benefit at Once

The pilot enabled shoppers to earn the entire benefit at once, rather than having to visit the market weekly, which allowed more shoppers, particularly those experiencing mobility challenges, to obtain the full monthly benefit amount. Farmers’ market staff reported some challenges associated with this feature, such as difficulty dispensing larger than usual amounts of scrip (which typically come in only USD 1 increments) and running out of scrip mid-market—challenges that could be relatively easily addressed if the pilot were to be expanded.

Few studies have examined the impact of monthly vs. weekly incentive maximums on shoppers and staff. The Healthy Incentive Program (HIP) implemented in Massachusetts offers shoppers a monthly, rather than per-visit, incentive maximum, and evaluations of HIP have shown the program to be highly utilized among SNAP shoppers and have associated the program with increases in produce purchases and consumption [24]. However, HIP evaluations did not specifically address customer or staff perceptions of the monthly vs. per-visit maximum, despite many HIP farmers’ markets previously offering per-visit maximums.

### 4.3. Delivery of Benefits as an Electronic Rebate

The pilot provided the supplemental benefit as an electronic rebate, i.e., a credit applied directly to shoppers’ EBT cards, rather than physical scrip, as is typical of traditional CNIP. Electronic rebates can be beneficial for shoppers, as they are typically automatically and seamlessly applied at the point of sale, with no extra effort required of the shopper. For this reason, “automatic discounts” have been found to have higher redemption rates compared with “physical” incentives, such as scrip or coupons [11].

However, electronic rebates can be challenging to implement at farmers’ markets, where shoppers must typically exchange EBT for physical scrip at a manager’s booth before shopping, as vendors are often unable to directly apply incentives at the time of sale. This was the case for the pilot since shoppers were required to purchase fruits and vegetables at the market to earn the incentive and thus needed to obtain scrip to make those purchases. Since shoppers were required to obtain scrip for the purchase of fruits and vegetables before shopping, they had to specify how much they intended to spend on produce in advance, which was challenging and felt invasive. Additionally, the requirement to obtain scrip before shopping contributed to long lines, which was challenging for shoppers and staff. Ultimately, the continued need for the use of scrip at pilot-participating farmers’ markets negated the “seamlessness” of the automatic rebate. This issue could potentially be addressed by providing individual vendors with the ability to process EBT transactions—and subsequently offer rebates on individual purchases—similar to the HIP [29]. This approach would likely require training produce vendors on how to use the technology and providing vendors with enough point-of-sale devices to process their EBT transactions [24]. If these issues were addressed, some benefits of automatic rebates may be more fully realized by shoppers and staff; however, the issue of shoppers choosing to spend the incentives earned at grocery stores and other retail outlets would not likely be impacted.

### 4.4. Ability for Shoppers to Redeem Benefits on All SNAP-Eligible Items Anywhere That Accepts SNAP

Perhaps the most significant feature of the pilot that differentiates it from traditional CNIP is the ability to redeem benefits anywhere that accepts SNAP, including grocery stores. Virtually all shoppers appreciated this feature, and most reported using it, noting that they could purchase a wider variety of foods, often at lower prices than at the farmers’ market. This finding aligns with research demonstrating that grocery stores typically offer shoppers a broader array of food options, many at lower prices and consistently with more accessible hours than farmers’ markets [8,9]. Farmers’ market staff also expressed concerns that shoppers redeeming benefits at grocery stores would negatively impact farmers’ market sales and local growers. There is little research examining the impact of allowing shoppers to redeem nutrition incentive benefits earned at farmers’ markets at grocery stores, as most nutrition incentive programs do not offer this feature [11].

### 4.5. Recommendations

Shoppers and farmers’ market staff offered several issues driving a need for improving program design and implementation. Both shoppers and staff cited a need to reduce long lines, which could be accomplished by means of explaining the program more clearly and concisely, particularly to non-English speakers, as well as more point-of-sale terminals to process EBT transactions. Shoppers also struggled with logistical issues related to the denomination of tokens, so larger-value tokens and more tokens being available could both speed up the process and facilitate the shopping experience. Farmers’ market staff recommended efforts to reduce reporting requirements and cited concerns about potential negative impacts on farmers’ markets associated with this model. Paying careful attention to the burden and benefit of this model to farmers’ markets will be critical to avoid negative unintended consequences.

Program sustainability must also be considered moving forward. The pilot ended in March 2024, when CDSS reported that program demand outpaced funding [30]. Future efforts should include sufficient funding to meet anticipated high consumer demand. Further, safety-net participants generally appreciate access to consistent support rather than constantly changing programs and funding [31]. Thus, scaling up a program such as this pilot should be carried out only when the resources are likely to be available to support the program for a meaningful period so that people with limited incomes can predict the benefits to which they have access and plan their finances accordingly.

### 4.6. Study Strengths and Limitations

The strengths of this study include the qualitative research design, which allowed us to capture rich and nuanced insights into participants’ lived experiences with the pilot program.

This study has several limitations. Participants were recruited based on interest, which may have resulted in self-selection bias. Additionally, a small number of shopper participants were recruited by market managers, who may have referred individuals with certain perceptions of the program. Participants were required to use technology (text message or e-mail and Zoom) and speak English or Spanish to engage in this study, which could have further limited participation and created bias.

### 4.7. Future Research

This study assessed the implementation of a pilot program in farmers’ markets that provided a new model of distributing SNAP supplemental benefits to shoppers. Future research should assess the impact of the program on farmers’ market sales and on local agriculture and sustainable food systems more broadly, given that supplemental benefits earned are not required to be spent at farmers’ markets. Exploring where shoppers spend the incentive, what they purchase, and impacts on diet and household food security would provide important insights. Future research should also assess the impact on farmers’ markets of adding the opportunity for SNAP shoppers to earn supplemental benefits for fruit and vegetable purchases at grocery stores.

## 5. Conclusions

The findings of this evaluation of the California Fruit and Vegetable EBT Pilot Project demonstrated that SNAP shoppers appreciated most aspects of the program and farmers’ market staff supported the enhanced purchasing power of SNAP shoppers; however, staff reported numerous challenges, and four out of six farmers’ markets included in this study ultimately discontinued participation in the pilot. The study findings indicate there are tradeoffs to expanding this supplemental benefit program model in California and nationally.

## Figures and Tables

**Table 1:** Questions asked in focus groups conducted with Supplemental Nutrition Assistance Program shoppers participating in the Fruit and Vegetable Electronic Benefit Transfer Pilot Project in California, 2023.

1. When did you start using Market Match at the farmers’ market?
2. Have you noticed a change in the last six months in how the Market Match program works at the farmers’ market where you shop? Can you talk about how the program used to work and how that is different from how it works now?
3. What do you like about the Fruit and Vegetable Pilot program? What do you not like?
4. How easy or difficult was it for you to understand how the Fruit and Vegetable Pilot program works?
5. How often do you all shop at a farmers’ market that offers the Fruit and Vegetable Pilot program? Has the Fruit and Vegetable Pilot program changed how often you shop at farmers’ markets?
6. Where do you usually spend the incentive or extra dollars that you receive through the pilot program?
7. Can you tell me about the types of items you purchase with the extra incentive dollars? Has the Fruit and Vegetable Pilot program affected the way you use the extra dollars that can be spent on any CalFresh ^1^-eligible items?
8. Can you talk about whether the Fruit and Vegetable Pilot program has affected how much of your CalFresh benefits you spend at the farmers’ market?
9. What changes have there been in the amount of fruits and vegetables that you and your family eat as a result of the Fruit and Vegetable Pilot program?
10. What changes have there been in the types of fruits and vegetables you and your family eat as a result of the Fruit and Vegetable Pilot program?
11. What changes have there been in the amount of other types of food you and your family have each month/week as a result of the Fruit and Vegetable Pilot program?
12. How about the overall amount of money you and your family have to spend each month on food?
13. What recommendations do you have for improving the way the Fruit and Vegetable Pilot program works?
14. For those of you that have used both the old Market Match and the new Fruit and Vegetable Pilot programs, which way do you like better? Why?

^1^ CalFresh is the term used for the Supplemental Nutrition Assistance Program in California.

**Table 2:** Questions asked in focus groups with farmers’ market staff at markets participating in the Fruit and Vegetable Electronic Benefit Transfer Pilot Project in California, 2023.

1. Can you describe your understanding of the new approach to the Market Match program—or the “pilot program”? For example, how would you describe the program to a new shopper at the market? Could you describe how it differs from the way the program used to work before the pilot program started?
2. What do you like about the pilot program approach to Market Match? What do you dislike about it?
3. How easy or difficult has it been for you to understand the pilot program approach to Market Match?
4. How easy or difficult has it been to explain the pilot program to shoppers?
5. How different has it been to understand and explain the new approach compared to the old approach?
6. Please describe any benefits you feel the pilot program provides to you or to your vendors or shoppers.
7. Please describe any challenges you feel the pilot program introduces to you or to your vendors or shoppers.
8. What feedback, if any, have you heard from CalFresh ^1^ shoppers and market vendors about the pilot program?
9. Do you notice any changes in how CalFresh shoppers use the pilot program compared to the way they used the old approach to Market Match?
10. Thinking about the old approach to Market Match and the pilot program, which do you prefer? Why?
11. What recommendations do you have for improving the pilot program?

^1^ CalFresh is the term used for the Supplemental Nutrition Assistance Program in California.

**Table 3:** Description of focus groups conducted with Supplemental Nutrition Assistance Program shoppers participating in the Fruit and Vegetable Electronic Benefit Transfer Pilot Project in California, 2023.

Group Number	Date	Market(s) Represented	Number of Participants	Focus Group Language
1	22 June 2023	Northern California Market 1	2	English
2	23 June 2023	Northern California Market 1	1	English
3	23 June 2023	Northern California Market 2	7	English
4	29 June 2023	Southern California Market 1	10	English
5	20 July 2023	Northern California Market 3	9	English
6	10 August 2023	Southern California Market 1	2	English
		Southern California Market 2	1	
		Southern California Market 3	2	
		Northern California Market 3	2	
7	10 August 2023	Northern California Market 2	4	Spanish

**Table 4:** Demographics of participants of focus groups conducted with Supplemental Nutrition Assistance Program shoppers participating in the Fruit and Vegetable Electronic Benefit Transfer Pilot Project in California, 2023 (*n* = 40).

Age	n (%)
18–30	10 (25.0)
31–50	18 (45.0)
51–70	10 (25.0)
71+	2 (5.0)
**Gender (multiple responses)**	**n (%)**
Woman	26 (65.0)
Man	11 (27.5)
Other/non-binary	4 (10.0)
Prefer not to answer	1 (2.5)
**Race/Ethnicity (multiple responses)**	**n (%)**
American Indian or Alaska Native	1 (2.5)
Asian	10 (25.0)
Black or African American	2 (5.0)
Hispanic or Latinx	10 (25.0)
Native Hawaiian or Pacific Islander	1 (2.5)
White	17 (42.5)
Other	3 (7.5)
Prefer not to answer	1 (2.5)
**Highest level of education**	**n (%)**
Some high school or less	3 (7.5)
High school graduate, G.E.D, vocational training certificate or license	5 (12.5)
Some college or Associate’s degree	12 (30.0)
Bachelor’s degree or higher	18 (45.0)
Prefer not to answer	1 (2.5)
Do not know	1 (2.5)
**Household Income**	**n (%)**
Less than USD 30,000	29 (72.5)
USD 30,000–USD 39,999	3 (7.5)
USD 40,000–USD 49,999	1 (2.5)
USD 50,000 or more	1 (2.5)
Prefer not to answer	2 (5.0)
Do not know	4 (10.0)
**Monthly Supplemental Nutrition Assistance Program (SNAP) benefit amount (n = 33)**	**Mean (Range)**
Monthly SNAP amount (USD)	USD 343 (USD 23–USD 1200)

**Table 5:** Number of staff from farmers’ markets implementing the Fruit and Vegetable Electronic Benefit Transfer Pilot Project participating in staff focus groups in California, 2023.

Market	Number of Participating Staff
Southern California Market 1	5
Southern California Market 2	3
Southern California Market 3	1
Northern California Markets 1 and 2 ^1^	3
Northern California Market 3	2
Total	14

^1^ These markets were operated by the same staff.

**Table 6:** Key findings and supporting quotes from focus groups conducted with Supplemental Nutrition Assistance Program shoppers participating in the Fruit and Vegetable Electronic Benefit Transfer Pilot Project and from farmers’ market staff at farmers’ markets implementing the Fruit and Vegetable Electronic Benefit Transfer Pilot Project in California, 2023.

Key Findings from Shopper Focus Groups
**Shopper Key Finding 1: Shoppers appreciated being able to receive the entire monthly benefit during one shopping trip.**
*I like this program because they give us the money in one lump sum and that allows me to buy the most I can in just one purchase*.—Spanish-speaking shopper, Southern California market *I’m disabled…I try to get [to the farmers’ market] but if I can’t, then on the plus side, at least I know that I can still use [all] my Market Match. And so that’s the positive, as opposed to the way that it was designed before where I would lose out*.—English-speaking shopper, Northern California market
**Shopper Key Finding 2: Shoppers liked being able to keep the USD 60 benefit on their EBT ^1^ card.**
*I think when you have 60 extra dollars that’s not coming off your card, it’s always going to be helpful. And compared to, I guess, the previous Market Match, right? You have to take out $10 to get $10, right? So free money to go towards extra groceries is always helpful*.—English-speaking shopper, Northern California market *I think the whole grocery store thing is great. Because you can go in and get whatever you want, whenever you want*.—English-speaking shopper, Northern California market
**Shopper Key Finding 3: Most shoppers reported spending the extra USD 60 on staple items at grocery stores; however, some shoppers reported spending it at the farmers’ market.**
*What I do with that extra money, either I use it at another farmers’ market, or honestly, I buy stuff that I can’t purchase at the farmers’ market, like milk*.—English-speaking shopper, Southern California market *So I use mine in the grocery stores for my normal groceries. It just gives me more buying power on my [EBT] card when I go to the grocery store.—*English-speaking shopper, Northern California market *I buy everything I can [at the farmers’ market]; fruits, vegetables, eggs. It all gets spent.—*Spanish-speaking shopper, Southern California market
**Key Findings from Shopper Focus Groups and Staff Focus Groups**
**Shopper and Staff Key Finding 1: Shoppers and staff appreciated the ability of shoppers to obtain more benefits with the pilot.**
*That’s a no brainer, duh. $60 [per month] versus $10 [per week]? Really?—*English-speaking shopper, Northern California market *The shoppers who especially need the support are getting more financial incentives and the ability to purchase these healthier fruits and vegetables... And yeah, that’s super, super positive.—*Staff member, Northern California market
**Shopper and Staff Key Finding 2: Shoppers and staff felt that the pilot attracted new SNAP ^2^ shoppers to pilot-participating farmers’ markets.**
*I stumbled upon this program and I saw that it’s only at limited farmers’ markets, so I started going to this farmers’ market once a month to participate in this program.—*English-speaking shopper, Southern California market *The pilot program has brought new people, new shoppers. There’s people that just head over there because of a pilot program. And it does seem busier.—*Staff member, Southern California market *I think I have seen an increase in participation. So it does seem that [the pilot program] is reaching out to more of the community and people are coming out.—*Staff member, Southern California market
**Shopper and Staff Key Finding 3: Shoppers and staff reported that the pilot was difficult to understand.**
*I experienced a learning curve for sure… I think it kind of seems like for everyone it took a couple tries to fully understand what was happening.—*English-speaking shopper, Southern California market *What I don’t like is that [the program] is so confusing, and it’s still confusing for me.—*Staff member, Southern California market
**Shopper and Staff Key Finding 4: Shoppers and staff reported that the pilot created long lines at participating farmers’ markets.**
*I’ve seen like 10 people in line sometimes and it goes really, really slow, because [the staff are] spending so much time talking and explaining the details. —*English-speaking shopper, Northern California market *[Shoppers] don’t like being in the line, they don’t like how long it’s taking… Sometimes it can get a little bit tense, when you can literally see somebody’s body language change from minute 5 to minute 25 of being in a line. It just feels particularly inhumane to have people on an 80-degree day standing with no sun cover.—*Staff member, Northern California market *We did lose regulars who would normally come every week to spend $15 to get the $15 due to the line that we are getting now. And yeah, a lot of our seniors are getting frustrated about the wait time.—*Staff member, Southern California market
**Shopper and Staff Key Finding 5: Shoppers and staff reported technological and logistical issues when operating and using the pilot.**
*I’ve had a couple of times, some farmers markets where they’ll double charge me. And I used to try to call the CalFresh ^3^ customer line. And they would never reverse it. They would just say, ‘no, it’s correct.’—*English-speaking shopper, Southern California market *Nothing could prepare me for the scenarios and technology problems that have popped up, where you have upset customers over what’s being reflected on their receipt, and they’re thinking that they’re being dinged somehow or that we’re taking money out of their cards, things like that…. We…didn’t anticipate that there would be so many quirks with the technology and what reads on people’s EBT balances and receipts and things like that.—*Staff member, Southern California market *Keeping up with the amount of transactions we’re doing now is near impossible. Like we’re bringing close to $10,000 worth of coins and we’re running out halfway through the market….. We’re having to go trade, chase them down from vendors who have yet to turn them in to us for their credits.—*Staff member, Northern California market
**Key Findings from Staff Focus Groups**
**Staff Key Finding 1: Staff appreciated that shoppers had expanded shopping options; however, some expressed concerns that shoppers were spending fewer EBT dollars at the market.**
*The customers really seem to like the program, and especially the part about being able to put it on their card, still getting fresh fruits and veggies, and then being able to shop at the store with their CalFresh benefits as well.—*Staff member, Northern California market *I like the fact that people do have more funds that they can use anywhere that they need it. What I don’t like is that it means in a way that the farmers’ markets are subsidizing [grocery stores] and all the other places where people go to get food, and it should be the other way around.—*Staff member, Southern California market
**Staff Key Finding 2: Staff appreciated that shoppers had increased benefits, with some noting they were able to offer the full benefit to shoppers with a SNAP balance below USD 60.**
*If I had a customer that has a minimum of $23 a month… I’ll keep doing the transaction where I give them $5 of fruit and veggie vouchers, redeem it, put it back on their card and keep doing that so they have a returning balance until they hit the $60.—*Staff member, Northern California market *I love the fact that we’re able to stretch out benefits for some of our customers, especially those that are coming in towards the end of the month, and just have the lowest balance… The fact that we found this out has been extremely helpful. I mean, we had three [shoppers] this past weekend, I believe, that we did that for, and they were so grateful. So I love that we’re able to help them out in that in that way.—*Staff member, Southern California market
**Staff Key Finding 3: Staff expressed concerns that the pilot required more—and more highly trained—staff compared to traditional CNIP ^4^.**
*It costs us in personnel almost three times as much to administer this program as the original Market Match… We’ve got five people just on the EBT portion.—*Staff member, Southern California market *I think if they expand [the pilot] to all of our sites, we’d have staff who definitely would not be up for the challenge. They would probably not be willing to be managing it on their own at a site. Because it does take someone who’s pretty skilled, who has a lot of patience.—*Staff member, Northern California market
**Staff Key Finding 4: Staff reported that the pilot increased their workload and diminished their opportunities to engage with shoppers.**
*The [pilot] program isn’t giving us enough time to make that one-on-one connection with shoppers.—*Staff member, Southern California market *Keeping up with the amount of transactions we’re doing now is near impossible... It just becomes such a different experience than what [staff] signed up for and what we recruited them to do. It’s an endless cycle of never being able to breathe.—*Staff member, Northern California market *The backend paperwork is definitely easier with [the traditional] Market Match and takes much less time.—*Staff member, Northern California market
**Staff Key Finding 5: Staff reported concerns regarding transparency with the EBT transaction and invasive questions regarding fruit and vegetable purchases.**
*In the beginning, when we were trying to understand the program, we would just ask, ‘What are you buying today? Are you here just for fruits and veggies?’ And I would notice [shoppers] were a little apprehensive, like, ‘Why are you asking me that?’… They don’t want to share that information.—*Staff member, Southern California market *The process of just figuring out how you’re going to get [a shopper] to buy in and feel like they can trust the system, you know, taking $60 off of their card, and just hoping it comes back on is a really big thing to ask for a lot of people.—*Staff member, Northern California market
**Staff Key Finding 6: Staff reported heightened challenges among shoppers with limited English.**
*I think one of my biggest challenges personally at the market I run is that I have a lot of language barriers…. I have a lot of shoppers who speak Russian, Arabic, Farsi, Dari. And so the language barrier there has been a little bit challenging.—*Staff member, Northern California market *We have different languages that we have to communicate with people through. So that’s also been a struggle.—*Staff member, Southern California market

^1^ EBT = Electronic Benefit Transfer. ^2^ SNAP = Supplemental Nutrition Assistance Program. ^3^ CalFresh is the term used for the Supplemental Nutrition Assistance Program in California. ^4^ CNIP = California Nutrition Incentive Program.

## Data Availability

The original contributions presented in the study are included in the article, further inquiries can be directed to the corresponding author.
